# Prostatic schwannoma discovered after laparoscopic radical prostatectomy: A case report with literature review

**DOI:** 10.1002/iju5.12667

**Published:** 2023-11-14

**Authors:** Tanan Bejrananda, Nattawit Jakjaroenrit

**Affiliations:** ^1^ Division of Urology, Department of Surgery Prince of Songkla University Songkhla Thailand

**Keywords:** case report, laparoscopic radical prostatectomy, prostate, schwannoma

## Abstract

**Background:**

Schwannomas originating in the prostate are extremely rare. We present a case of prostatic schwannoma in a 66‐year‐old male with lower urinary tract symptoms. Preoperative evaluation revealed a prostatic mass, and the definitive diagnosis was made through laparoscopic radical prostatectomy.

**Case presentation:**

A 66‐year‐old male presented with persistent lower urinary tract symptoms for 5 years and a prostate‐specific antigen level of 0.63 ng/mL. MRI showed a well‐defined solid cystic mass in the posterolateral basal right peripheral zone, causing superior displacement of the right seminal vesicle. Laparoscopic radical prostatectomy was performed, confirming a periprostatic schwannoma.

**Conclusion:**

This case highlights the rarity of prostatic schwannomas and their association with lower urinary tract symptoms. MRI plays a crucial role in identifying prostatic masses, while laparoscopic radical prostatectomy can serve as a diagnostic and therapeutic approach for prostatic schwannomas. Increased awareness of this rare entity is essential for accurate diagnosis and optimal management.

**Abbreviations & Acronyms**
BPH: benign prostatic hyperplasia
CT: computed tomography
DRE: digital rectal examination
LUTS: lower urinary tract symptoms
mpMRI: multiparametric magnetic resonance imaging
MRI: magnetic resonance imaging
PSA: prostate‐specific antigen
TRUS: transrectal ultrasound

**Keynote message**

Prostatic schwannomas are extremely rare tumors of the prostate, often presenting with lower urinary tract symptoms. Imaging techniques such as MRI can be valuable in identifying these masses. Laparoscopic radical prostatectomy serves as an effective diagnostic and therapeutic approach for prostatic schwannomas, emphasizing the importance of awareness and accurate management of this uncommon condition.

## Background

Schwannomas, which are composed of Schwann cells and arise from peripheral nerves, are typically benign tumors. While they are commonly solitary masses found in soft tissues or spinal nerve roots,^1^ schwannomas affecting the male genital system are infrequent and often managed surgically. Prostatic schwannomas, specifically, are extremely rare, with only a few cases documented in association with neurofibromatosis and even fewer occurring sporadically.^2^, ^3^, ^4^, ^5^, ^6^, ^7^, ^8^ In this report, we present a unique case of prostatic schwannoma identified during the evaluation of a prostatic mass, ultimately confirmed through laparoscopic radical prostatectomy.

## Case presentation

**Fig. 1:**
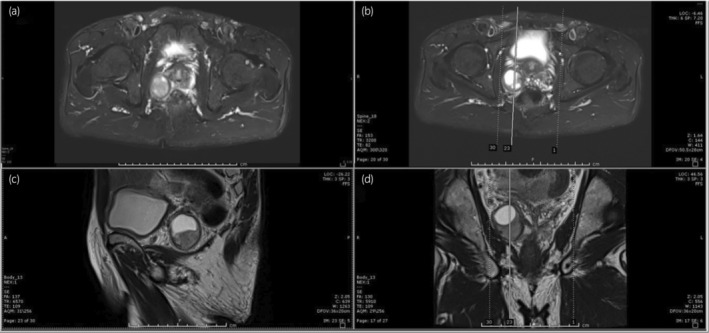
Coronal (a) and axial (b) T2‐weighted MRI of PI‐RADS 4 adenocarcinoma. Coronal (c) and axial (d) T2‐weighted MRI of periprostatic cystic lesion.

**Fig. 2:**
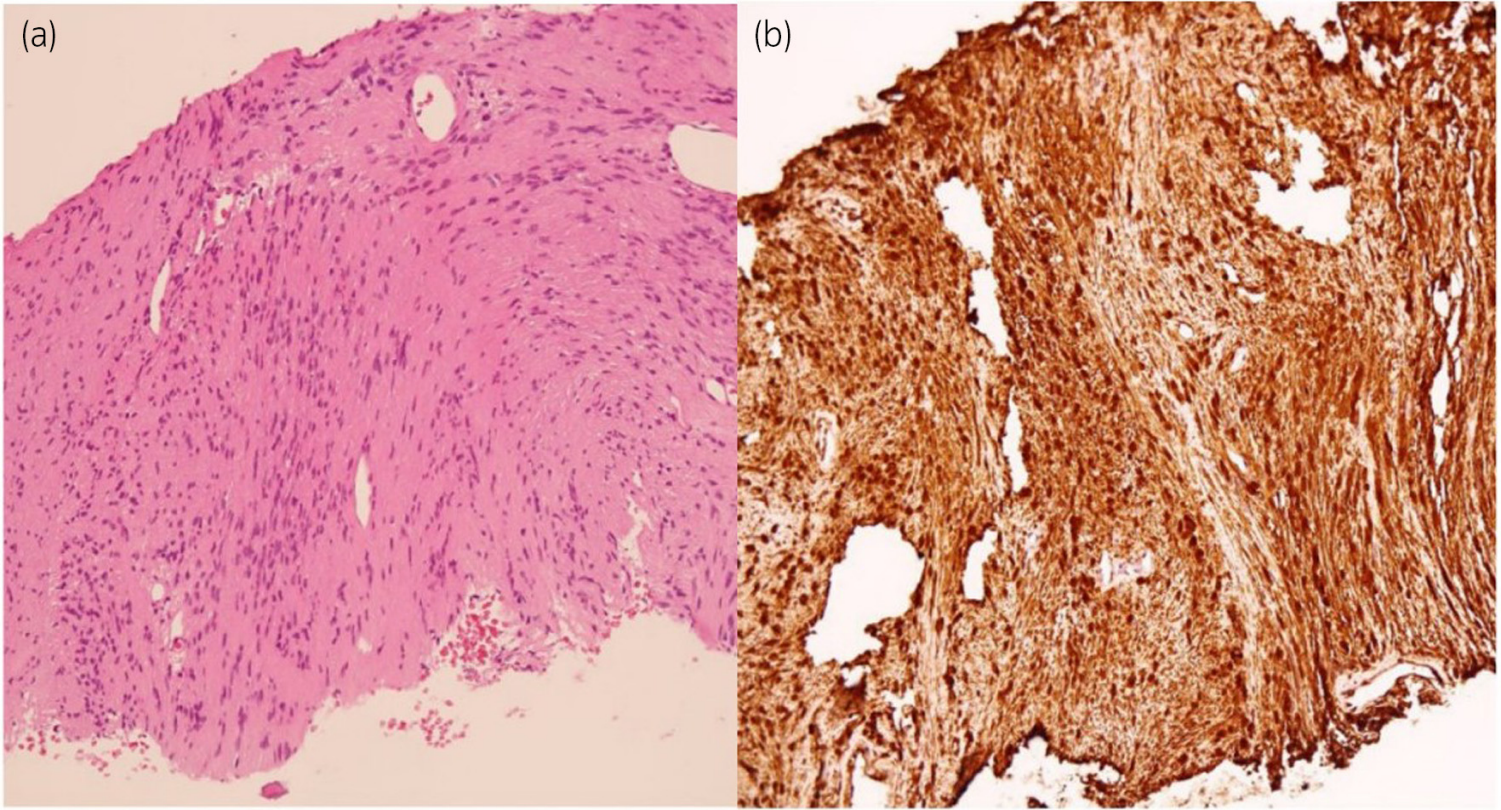
(a) Histopathological finding with hematoxylin and eosin stain (×200) revealed spindle‐shaped cells arranged in fascicle pattern. (b) The cells were strongly positively stained after immunostaining for S100 (×200).

A 66‐year‐old male patient, who had been under the care of a local urologist for LUTS and had a normal PSA level (0.63 ng/mL), underwent a comprehensive evaluation. Physical examination and metabolic panel results were unremarkable. DRE revealed moderate prostate enlargement with a rubbery consistency but no palpable hard nodule. Subsequent multiparametric MRI of the prostate demonstrated a solid cystic mass in the right periprostatic space, exerting mass effect on the right peripheral zone of the prostate gland and right seminal vesicle, measuring 3.0 × 3.1 × 3.3 cm. The imaging findings suggested a right periprostatic mass with a solid cystic stromal tumor, possibly leiomyosarcoma or rhabdomyosarcoma (Fig. 1). TRUS‐guided prostate biopsy was performed, revealing BPH and ruling out cancer. One year later, the decision to perform a repeat MRI was made because the initial biopsy, while confirming BPH, did not align with the MRI findings, which indicated a solid cystic mass with characteristics that raised suspicion of a different pathology, possibly a sarcoma. The repeat MRI was conducted to monitor the progression of this mass and gather more information for treatment planning, follow‐up MRI showed slight growth of the well‐defined solid cystic mass originating from the posterolateral basal right peripheral zone of the prostate gland, displacing the right seminal vesicle. The decision to proceed with laparoscopic radical prostatectomy in this case was primarily based on the discrepancy between the MRI findings and the initial biopsy, which suggested a solid cystic stromal tumor, possibly leiomyosarcoma or rhabdomyosarcoma. This posed diagnostic uncertainty, and surgical intervention was deemed necessary to obtain a definitive histopathologic diagnosis. Preoperative differential diagnoses included stromal tumor with unknown malignant potential suspected leiomyosarcoma. Biopsy in this case was performed using TRUS guidance. The patient underwent laparoscopic radical prostatectomy, during which the cystic lesion was successfully removed. Gross examination of the prostate revealed a well‐defined yellowish cut surface with a size of 3.5 × 3.5 cm, comprising a solid component and clear fluid cystic component predominantly involving the right posterolateral base. Histopathological examination, including immunostaining for S100 (Fig. 2), and negative for SMA, desmin, and CD34, confirmed the diagnosis of prostatic schwannoma. The patient's postoperative continence and erectile function outcomes were full recovery functions after 6 months.

## Discussion

This report represents the eighth documented case of prostatic schwannoma and the second case of periprostatic schwannoma. Given the broad range of potential prostatic and periprostatic lesions, including neoplastic, inflammatory, and congenital processes, the differential diagnosis can be extensive, ranging from benign cystic lesions to periprostatic stromal sarcomas. Due to the wide variety of diagnoses, prognosis and treatment approaches can vary significantly. Imaging findings alone are insufficient for a definitive diagnosis, necessitating histopathologic examination. Although uncommon, schwannoma should be considered when encountering a well‐defined, hypoechoic, or cystic lesion on prostatic MRI or ultrasound. Sporadic schwannomas in the genitourinary system, including the penis, spermatic cord, testis, or prostate, remain exceedingly rare.^5^ The role of imaging in this case was pivotal for identifying and characterizing the prostatic mass. Imaging, specifically multiparametric mpMRI, played a crucial role in locating the mass, determining its size, and assessing its relationship with adjacent structures. The imaging findings guided further diagnostic and treatment decisions. In cases where neurofibromatosis is present, the urinary bladder is the most frequently affected genitourinary organ, typically manifesting as neurofibroma.^1^ The patient in this case presented with a prostatic schwannoma, without any prior history of neurofibromatosis. The diagnosis was confirmed through the evaluation and examination of the schwannoma. Additionally, a literature review was conducted, highlighting the existing knowledge and reported cases of prostatic schwannomas, we have summarized the treatment strategies of the cases published in the literature related to prostatic schwannomas. We highlight the variations in management approaches, including surgical excision and the importance of histopathologic examination for diagnosis. (Table 1).

**Table 1:** Literature review of sporadic prostatic schwannomas

Case	Year	Age	Present illness	Physical exam	Schwannoma location	Treatment	Outcome	References
1	1995	55	Four years of obstructive symptoms	Benign enlargement of prostate, PSA n/a	Intraprostatic, size n/a	Exploratory laparotomy	NED at 17 months postoperatively	Rane *et al*.^2^
2	2002	65	Asymptomatic	No palpable prostate nodule, PSA 2.4 ng/mL	Intraprostatic, peripheral zone, 1.2 cm	Observation	Unchanged disease 1‐year postobservation	Francica *et al*.^3^
3	2003	65	Painful hematuria, obstructive voiding	Enlarged, tender prostate, PSA 1.8 ng/mL	Intraprostatic, left lobe, 7 cm	Transvesical suprapubic prostatectomy	NED 2 years at time of publication	Jiang R *et al*.^4^
4	2016	64	Severe LUTS	Grade 3‐enlarged prostate. PSA 2.1 ng/mL	Intraprostatic, size n/a	Transvesical suprapubic prostatectomy	NED 18 months postoperatively	Üçer *et al*.^5^
5	2020	60	Mild LUTS	Mild enlarge prostate. PSA 4.84 ng/mL	Periprostatic cystic lesion, size 3.3 × 3.5 × 3.4 cm	Prostatectomy	NED 2 years at the time of publication	Dietrick *et al*.^6^
6	2021	67	LUTS consisting of increased frequency of urination and nocturia	Mild enlarge prostate. PSA 12.2 ng/mL	Intraprostatic, both lobe, size 5 cm in right and 1.6 cm in left	Robotic‐assisted laparoscopic radical prostatectomy	NED 1 year at the time of publication.	Tan *et al*.^7^
8	2021	65	Asymptomatic with PSA elevation	Enlarge prostate, hard nodule on the left side. PSA 12.6 ng/mL	Intraprostatic, left peripheral lobe, size 1.54 cm	Conservative treatment	6‐year follow‐up	Lai *et al*.^8^

In the presented cases of prostatic schwannoma, it is noted that only two cases had elevated PSA levels, with one of those cases being associated with prostate adenocarcinoma. This indicates that elevated PSA levels are not commonly observed in prostatic schwannomas, further distinguishing them from other prostate conditions.^8^

Indeed, in the reported cases of prostatic schwannomas, only one case had palpable nodules upon DRE, which is consistent with their mostly intraprostatic or periprostatic location. The specific location of the lesion in this case was peripheral, likely arising from the posterolateral neurovascular bundle, and was not palpable. LUTS were commonly observed across the reported cases, ranging from mild to severe. However, imaging remains a crucial investigation for further evaluation and treatment planning.

Diagnosing schwannomas in the genitourinary system, including the prostate, can be challenging due to their nonspecific clinical and radiologic features. While ultrasonography may serve as an initial diagnostic tool, CT or multiparametric mpMRI can provide more detailed information regarding the size, location, local involvement, and potential spread of the tumor. However, the definitive diagnosis is made through histopathologic and immunohistochemical examinations of biopsied or excised specimens. It is important to note that schwannomas are not responsive to radiation therapy or chemotherapy, and surgical excision remains the primary treatment approach.

## Conclusion

We reported the case of a periprostatic schwannoma discovered finding of prostate tumor and treatment with laparoscopic radical prostatectomy. Data from clinical presentation and imaging, definitive diagnoses of prostatic masses must rely on histopathology, as prostatic schwannomas comprise a diagnostic possibility and may alter clinical decision‐making.

## Author contributions

Tanan Bejrananda: Conceptualization; writing – original draft; writing – review and editing. Nattawit Jakjaroenrit: Conceptualization; writing – original draft.

## Conflict of interest

The authors declare that we have no competing interest.

## Approval of the research protocol by an Institutional Reviewer Board

Ethics approval and consent to participate Approval for this study was given by the Ethics Committee of the Songkanagarind hospital, Prince of Songkla University, Faculty of Medicine, Prince of Songkla University with reference number REC.66‐238‐10‐1.

## Informed consent

Written informed consent was obtained from the patient for publication of this case report and any accompanying images. A copy of the written consent is available for review by the Editor‐in‐Chief of this journal.

## Registry and the Registration No. of the study/trial

Not applicable.

## Funding information

Not applicable.

## Data Availability

Not applicable.
